# Decoding Gastric Cancer: Machine Learning Insights Into the Significance of COMMDs Family in Immunotherapy and Diagnosis

**DOI:** 10.7150/jca.94360

**Published:** 2024-05-11

**Authors:** Rong Wan, Lujuan Pan, Qi Wang, Guanliang Shen, RuoNan Guo, Yueqiu Qin, Xufeng Huang, Ruo Wang, Xin Fan

**Affiliations:** 1 Department of Gastrointestinal Surgery, Affiliated Hospital of Jiangsu University, Zhenjiang, China.; 2 Department of Gastroenterology, Affiliated Hospital of Youjiang Medical University for Nationalities, No.18 Zhongshan 2nd Road, Baise, Guangxi, China.; 3 Key Laboratory of Tumor Molecular Pathology of Baise, Guangxi, China.; 4 Department of Gastroenterology, Affiliated Hospital of Jiangsu University, Jiangsu University, Zhenjiang, China.; 5 Department of Laboratory, Affiliated Hospital of Jiangsu University, Jiangsu University, Zhenjiang, China.; 6 Faculty of Dentistry, University of Debrecen, Debrecen, Hungary.; 7 Ruijin Hospital, Shanghai Jiao Tong University School of Medicine, Shanghai, China.

**Keywords:** Gastric Cancer, Machine Learning, COMMDs Family, Immunotherapy, TME

## Abstract

Copper, an indispensable trace element for the human body, serves not only as a crucial auxiliary factor in redox reactions within the organism but also as a significant constituent of numerous key metabolic enzymes. The COMMD family plays a vital role in regulating copper at both the cellular and systemic levels, particularly in the realm of tumor research, an area notably deficient in gastric cancer investigations. With the advancement of precision medical techniques, individualized and precise screening and treatment have become paramount considerations in the contemporary medical landscape for gastric cancer therapy. In light of this, we meticulously scrutinized existing transcriptomic datasets for gastric cancer, validating the expression levels and prognostic value of COMMD family genes. Simultaneously, employing the ssGSEA algorithm, we devised the COMMDs score. Enrichment analysis, gene mutations, and clinical features were incorporated into the assessment of this score. Furthermore, we contextualized the COMMDs score within the framework of the immune microenvironment, evaluating the relationship between the COMMDs family and immune factors as well as immune cells. The results suggest a correlation between the COMMDs score and various immune-related features. Based on this foundation, multiple machine learning approaches indicated Logistic Regression, with a remarkable ROC of 0.972, as the optimal diagnostic model. To accentuate the translational medical value of the COMMDs family, we selected COMMD10 as a differential gene in gastric cancer for further validation. Functional experiments revealed a decline in the proliferative and migratory capabilities of gastric cancer cells upon silencing COMMD10. Additionally, through pathway intervention, we unveiled the PI3K-AKT pathway as a potential mechanism through which COMMD10 influences gastric cancer activity. In summary, our study affirms the prospective role of the COMMDs family as potential markers for the diagnosis and treatment of gastric cancer in the future.

## Introduction

Gastric cancer stands as one of the most prevalent malignant tumors globally, casting a profound burden on the worldwide healthcare system due to its insidious onset and low screening rates. According to the 2020 statistical data from the American Cancer Society, gastric cancer ranks as the fifth most common cancer worldwide and the fourth leading cause of cancer-related deaths. Surgical intervention has consistently served as the frontline therapeutic approach for both early and advanced stages of gastric cancer. In recent years, interventions such as pre-operative and post-operative neoadjuvant chemotherapies, and notably, targeted therapies and immunotherapies, have come into our purview, garnering positive feedback 1-4. It is precisely these advancements that underscore the increasing importance of meticulous target selection with the progress of precision medical technologies. Personalized and precise screening and treatment thus emerge as the paramount considerations in the framework of modern medical practices 5.

Within the framework of gastric cancer and, by extension, the entire paradigm of cancer treatment, the COMM domain (COMMD) proteins are assuming an increasingly prominent role in the current landscape of targeted therapy. The COMMDs gene family represents a recently discovered cohort characterized by a distinctive motif defined by its extreme carboxy-terminal (copper metabolism MURR1, or COMM domain). The hallmark of COMMD family members is a highly conserved C-terminal sequence, comprising 70-80 amino acids, known as the COMM domain. Currently, the structural intricacies of this domain remain elusive 6, 7. Comprising 10 members, this protein family has undergone extensive conservation throughout the course of evolution. Individually or collectively within a protein-protein interaction network (PPI), they participate in various eukaryotic biological processes. These encompass copper homeostasis, ion transport, signal transduction pathways, cell proliferation, invasion, migration, angiogenesis, inflammation, and immune infiltration 8-10. Their dysregulation may lead to immune disorders, copper metabolism disturbances, and errors in control, culminating in the initiation and progression of cancer.

COMMD1 stands out as a multifaceted regulatory protein within the COMMD family, representing a paradigmatic member. HIF, a transcription factor, has been substantiated to play a pivotal role in promoting tumor growth, survival, and invasion 11. Studies indicate that COMMD1, through direct binding to the amino-terminal of HIF-1α, impedes its dimerization with HIF-1β, subsequently hindering DNA binding and transcriptional activation. This inhibition curtails HIF-1-mediated gene expression, effectively suppressing tumor cell invasion 12. Moreover, the downregulation of COMMD1 has been associated with an escalation in NF-κB activation levels *in vivo*, accompanied by heightened inflammation and increased expression of stem cell-related genes 13. In the realm of hepatocellular carcinoma (LIHC), Fang *et al.* 10 discovered a correlation between the upregulation of COMMD2 and an unfavorable prognosis linked to immune cell infiltration. High expression levels of COMMD2 were significantly correlated with elevated PD1, PD-L1, and CTLA-4 levels in LIHC, suggesting that targeted inhibition of COMMD2 might enhance the efficacy of immune therapy in LIHC. Zheng 14 suggesting that targeted inhibition of COMMD2 might enhance the efficacy of immune therapy in LIHC. The low expression of COMMD3 can inhibit HCC angiogenesis by inhibiting HIF1α/VEGF/NF-κB pathway 15. COMMD3 deficiency enhances copper overload in breast cancer, and subsequently ATPB1/ATP7A can be upregulated due to copper poisoning, which can promote solid tumor growth 16. COMMD4 is highly expressed in non-small cell lung cancer, and siRNA-mediated depletion of COMMD4 significantly reduces the proliferation and viability of lung cancer cell lines after exposure to ionizing radiation and camptothecin-induced double-stranded DNA breaks 8. Additionally, in liver cancer cells, the silence of COMMD7 significantly inhibited cell proliferation. Silencing NF-κB further repressed COMMD7 expression, subsequently suppressing cell proliferation. An intricate positive feedback loop exists between COMMD7 and the NF-κB pathway. Furthermore, targeted overexpression of COMMD8 has been shown to promote proliferation and invasion in non-small cell lung cancer and hepatocellular carcinoma 17-19. COMMD9 has been identified to participate in the activation of TFDP1/E2F1. Reduced expression of COMMD9 inhibits TFDP1/E2F1 activation, concomitant with enhanced activation of the p53 signaling pathway. COMMD9 has the potential to stimulate proliferation, migration, G1/S phase transition of the cell cycle, while inhibiting cellular autophagy in non-small cell lung cancer (NSCLC) 20, 21. The COMMD10/NF-κB axis promotes intrinsic apoptosis by regulating the Bcl-2/Bax/caspase-9/3 pathway, as demonstrated in hepatocellular carcinoma 22. FMNL2 targets ubiquitin-mediated proteasomal degradation in COMMD10 CRC cells. COMMD10 targets the p65 NF-κB subunit and reduces its nuclear translocation, which leads to inactivation of the NF-κB pathway and inhibition of CRC invasion and metastasis 23. These findings collectively affirm a preliminary theory that COMMDs genes may exert cell-type-specific roles in the migration and metastasis of tumors. Further elucidating the genetic attributes of this entire family and their potential mechanisms in relation to gastric cancer could subtly influence the current landscape of overall therapeutic environments.

To this end, our research regards the known 10 genes of the COMMDs family as a cohesive entity, delving into their potential diagnostic and therapeutic value within gastric cancer. Specifically, we scrutinized the expression and prognosis of these family genes in gastric cancer. Furthermore, our focus extended to their immunological characteristics and genetic mutation information. The progression of these steps was facilitated by the application of our developed COMMDs scoring system. In conclusion, the evaluation of over 20 machine learning algorithms underscores the significant translational medical value of the current COMMDs family genes. Altogether, these findings hold the promise of contributing, to varying degrees, to the overall improvement of survival rates for future gastric cancer patients.

## Methods

### Data Collection and Compilation

We obtained standardized and log2-transformed RNA sequencing (RNA-seq) profiles and corresponding clinical information from the TCGA database. For the gastric cancer validation set, we downloaded data from the GEO database, which included the following datasets: GSE13861, GSE15459, GSE183136, GSE26253, GSE26899, GSE26901, GSE28541, GSE34942, GSE438749, GSE57303, GSE62254, GSE84426, GSE 84433, and GSE 84437. When necessary, the “anno probe” R package was utilized to visualize probe maps. Additionally, in cases requiring it, the “limma” R package was employed to calculate the average values for multiple probes 24. Gene alterations within the COMMD family were assessed using the Genomic Cancer Atlas (GSCA) database. A total of 50 hallmark pathways were obtained from Gene Set Enrichment Analysis (GSEA) in the Molecular Signatures Database (MSigDB) and analyzed as per the previously described methods.

### Acquisition of COMMDs Scores

We employed the single-sample Gene Set Enrichment Analysis (ssGSEA) algorithm, implemented using the “GSVA” R package, to calculate the COMMDs score for each patient. Each ssGSEA score reflects the degree to which the COMMD family genes are coordinately upregulated or downregulated in an individual sample 25. The specific formula used is in accordance with the methodology outlined in the previous study by Xie *et al.* 26.

### Selection of Evaluation Methods for Immune-Related Features

The current mainstream eight algorithms, including CiberSort-ABS, EPIC, MCP-Counte, Quantiseq, Xcell, CiberSort, Estimate, Timer, were utilized to calculate the feature landscape of COMMDs in the gastric cancer immune microenvironment. The calculation process was uniformly conducted using the R package “immunedeconv” 27. The processed expression profiles of the COMMD family were correlated with various immune modulators, including antigen presentation, immune inhibition, immune stimulation, chemokines, and receptors. The results were presented in the form of heatmaps.

### Prognostic Analysis of Different COMMD Family Genes

The “Survival” and “Survminer” R packages were employed for Kaplan-Meier (K-M) analysis of the overall survival (OS) associated with different COMMD family genes. The median was chosen as a categorical variable, and predictive efficacy was assessed using chi-square tests and Fisher's exact tests. This process included the analysis of the correlation between COMMDs score levels and clinical-pathological parameters such as grading, staging, gender, T staging, lymph node metastasis, and others.

### Enrichment Analysis Corresponding to COMMDs Scores

The “ClusterProfiler” package was utilized for gene set enrichment analysis (GSEA) methods, encompassing KEGG, GSEA-HALLMARKS, and other entries.

### Establishment of Machine Learning Models and Decision Curve Analysis

We evaluated 20 mainstream machine learning algorithms to identify the optimal diagnostic model for COMMD family genes with potential translational medical value. Detailed algorithm descriptions were outlined in previous research by Wang *et al.* 28. The evaluation metrics for the models primarily relied on the ROC curve, where the area under the curve (AUC) represented predictive capability. Additionally, parameters such as accuracy, precision, recall, and F1 score were included to ensure stringent standards. All mentioned curves were created using the “scikit-learn” Python package.

### Reagents

The reagents used in the study were procured as follows: RPMI-1640 and DMEM/F12 culture media (Shanghai Zhong Qiao Xin Zhou Biotechnology Co., Ltd.); premium fetal bovine serum (Sangon Biotech Co., Ltd.); CCK-8 assay kit (Vazyme Biotech Co., Ltd.); Transwell chambers (Corning Incorporated USA); antibodies for Akt (Phospho-Ser473 1:1000 Sangon Biotech Co., Ltd.), p-Akt (#4060, Ser 473, 1:1000 CST), mTOR (#7C10, 2983S 1:1000 CST), p-mTOR (#AF3308, Ser2448, 1:1000), p-PI3K (#AF3242, Tyr458, 1:1000), PIK3R1 (#A00318-1, 1:1000), and GAPDH (ab8245, 1:10000); AKT pathway activator SC79 (#CSN16878 Csnpharm).

### Cell Culture

Cell lines HGC-27 and AGS were obtained from the Cell Bank, Type Culture Collection, Chinese Academy of Sciences, Cell lines HS-746T and MKN-45 were obtained from Shanghai Zhong Qiao Xin Zhou Biotechnology, authenticated via STR profiling. HGC-27 cells were cultured in RPMI-1640 medium with 10% fetal bovine serum, while AGS cells were cultured in DMEM/F12 medium with 10% fetal bovine serum, HS-746T and MKN-45 were cultured in a dedicated medium. These cell lines were maintained in a 5% CO2 incubator at 37°C. Cells were passaged upon reaching 80%-90% confluence using 0.25% trypsin.

### Cell Transfection

Cells were seeded in 6-well plates one day before transfection, with a cell density of 30% to 50%. Three pairs of small interfering RNAs (siRNAs) were designed and tested for transfection efficiency using RT-PCR and western blot. The sequences were as follows: 5'-GCAGUGUCACUGAUAAAUGCATT-3', 5'-GAGAGCAGUUUCAGUGAAGAATT-3', and 5' GCAGCAGCAAUUAGAGAACAUTT-3'. COMMD10 was silenced in HGC-27 and AGS cells using Rfect (BAIDAI) according to the manufacturer's instructions.

### RT-PCR

For RT-PCR, cells from experimental and control groups of HGC-27 and AGS cell lines were cultured and collected to detect COMMD10 expression. Total RNA was extracted from cultured cells using the RNA-Quick Purification Kit (YiShan Biotech), and cDNA was synthesized using the cDNA reverse transcription kit (Vazyme Biotech Co., Ltd.). RT-qPCR reactions were performed using a QuantStudio real-time fluorescence quantitative PCR system with the following primer sequences: GAPDH-F: GTCAGTGGTGGACCTGACCT, GAPDH-R: GTTGCTGTAGCCAAATTCGTTGT; COMMD10-F: GCAGCAGCAATTAGAGAACATTCATC, COMMD10-R: AGAGTGAGCCATCTGAAGGTTAAGC.

### Cell Viability and Colony Formation

Two siRNA sequences showing significant knockdown efficiency according to RT-PCR results were chosen for cell viability experiments. COMMD10-silenced HGC-27 and AGS cells were seeded in 96-well plates at a density of 5000 cells per well, with five replicates per group. The cells were cultured for 0, 24, 48, and 72 hours, and cell viability was assessed using the CCK-8 assay (Vazyme Biotech Co., Ltd.). Absorbance at 450 nm was measured using a microplate reader. For colony formation, cells (2000 cells per well) were seeded in 6-well plates, cultured for 7-14 days, and then fixed with 0.4% paraformaldehyde for 30 minutes. After staining with 0.1% crystal violet for 15 minutes, images were captured using a camera.

### Transwell and Scratch Assay

Cells with a density of 25*104/ml were taken for Transwell and scratch assays. For Transwell, 200 μL of cell suspension was added to the upper chamber, and 600 μL of medium containing 10% FBS was added to the lower chamber for 24 hours. Subsequently, the cells on the bottom of the lower chamber were fixed with 4% paraformaldehyde, stained with 0.1% crystal violet, and counted under an optical microscope. For the scratch assay, transfected GC cells were evenly seeded in a 6-well plate and grown until confluent. A sterile 200 μL pipette tip was used to scratch the cells, and the scratch was photographed at 0 h, 24 h, and 48 h to calculate the cell migration rate.

### Pathway Rescue Experiment

The AKT pathway activator SC79 was used to selectively activate the AKT pathway in control and experimental groups of GC.

## Results

### The Expression Profiles of the COMMD Family Genes in Gastric Cancer

**Figure 1:**
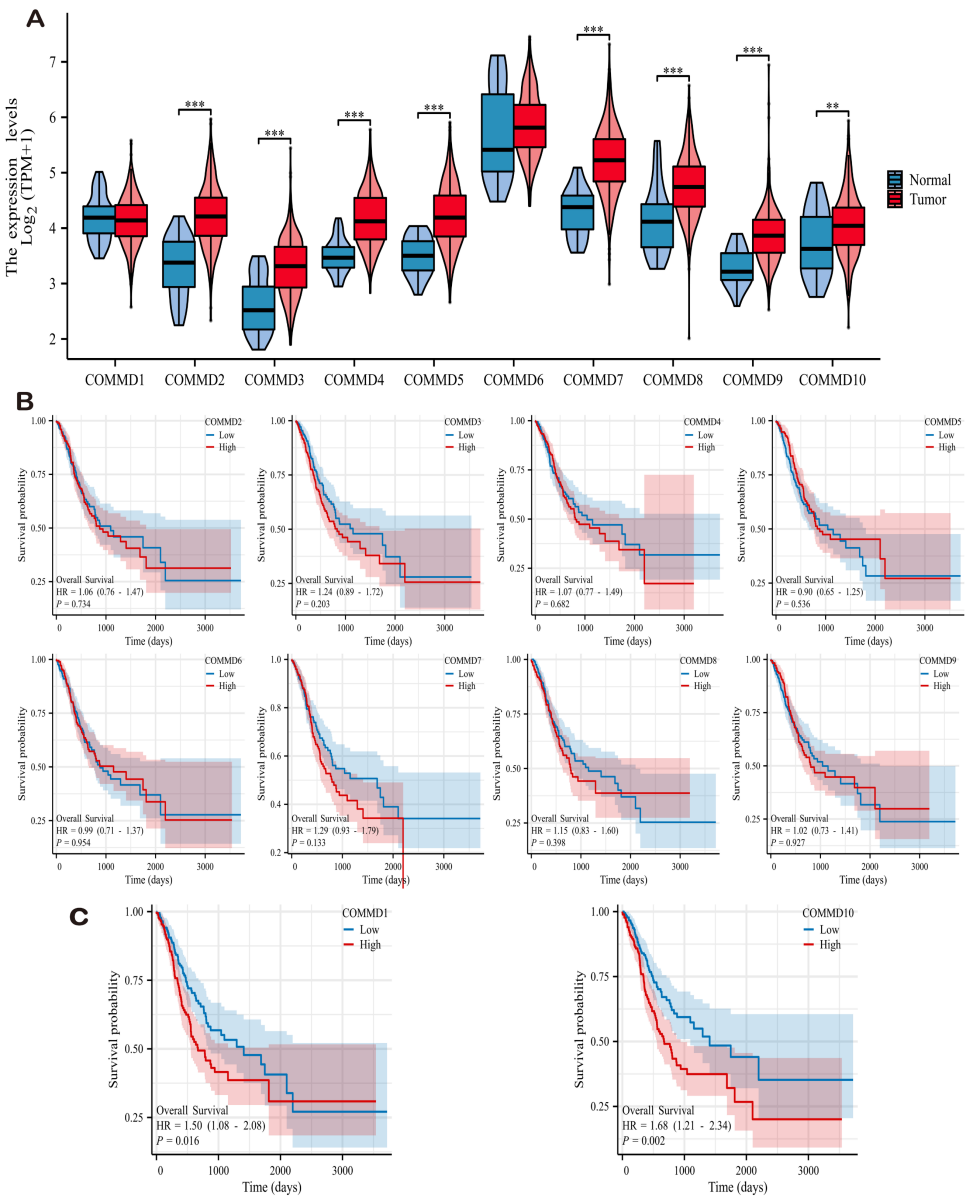
Differential Expression of COMMD Family Genes. (A) Violin plots depicting the differential expression of COMMD family genes in tumor and normal tissues based on TCGA dataset. (B) Impact of different COMMD genes on overall survival (OS) of gastric cancer patients.

Integrating transcriptomic data from gastric cancer patients in the TCGA and GTEx databases, we analyzed and investigated the expression levels of 10 COMMD family genes. As depicted in **(Figure 1A)**, except for COMMD1 and COMMD6, the remaining COMMD genes included in the study exhibited significant differential expression in gastric cancer. Furthermore, adjacent normal tissues revealed a marked downregulation of COMMD family genes, suggesting their potential role as tumor-promoting factors across various cancer types, the results are illustrated in **(Figure 1B)**. Kaplan-Meier (K-M) analysis was conducted for each COMMD gene in gastric cancer tissues, revealing COMMD1 and COMMD10 as significant high-risk factors for overall survival (OS) in gastric cancer patients **(Figure 1C)**.

### Establishment of COMMDs Score and Evaluation of Clinical Features

**Figure 2:**
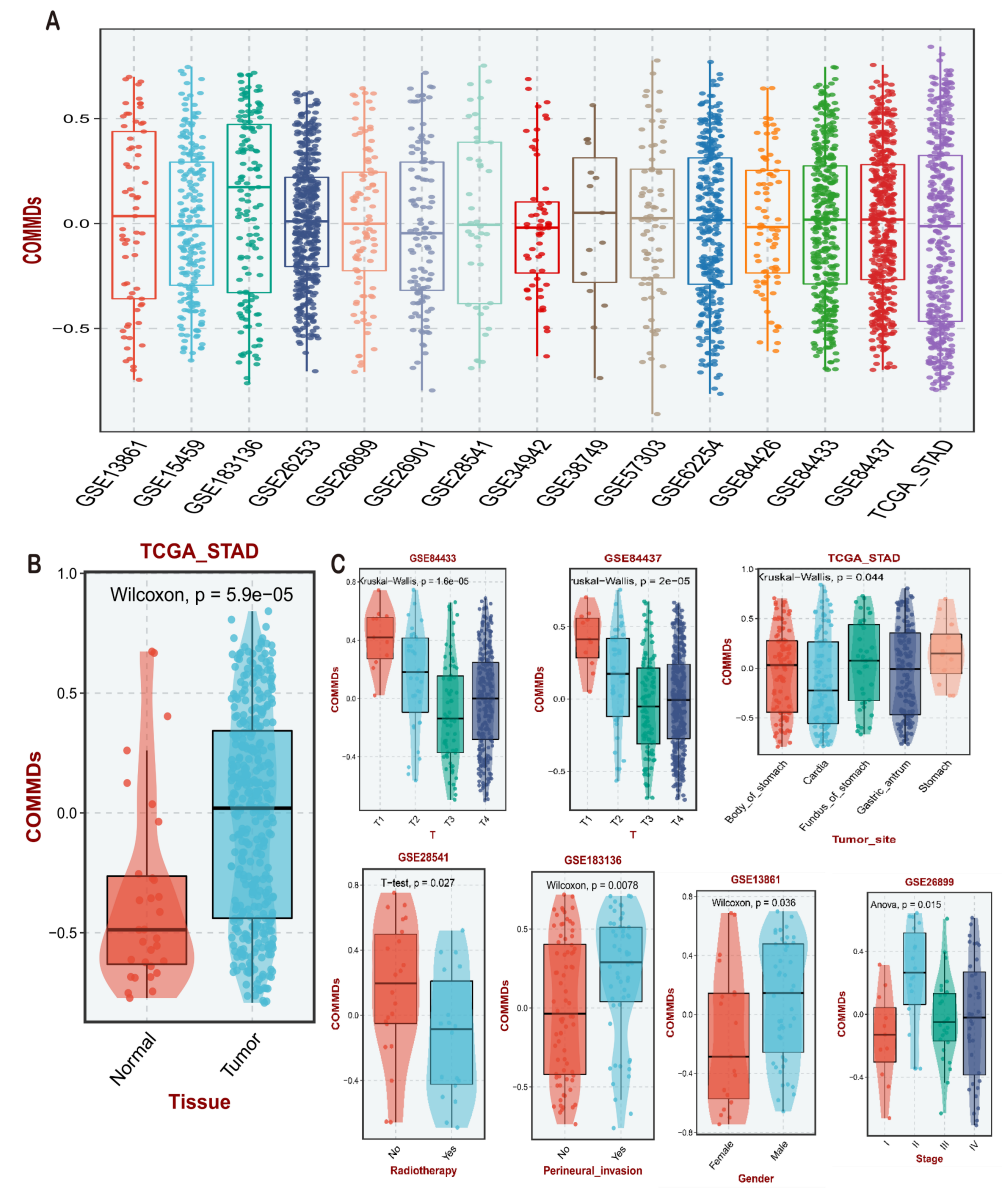
Establishment and Clinical Evaluation of COMMD Scores. (A) Construction of COMMD scores based on TCGA dataset and various GEO datasets, along with the distribution of COMMD scores. (B) Distribution of COMMD scores in tumor and non-tumor tissues. (C) Relationship between COMMD scores and various clinical characteristics of gastric cancer patients.

Using the single-sample gene set enrichment analysis (ssGSEA) algorithm in the TCGA STAD cohort, we established a scoring criterion defined as the COMMDs score. Validation was performed using publicly available gastric cancer transcriptomic datasets from the GEO database. The scoring results are presented in **(Figure 2A)**, demonstrating a relatively uniform distribution of COMMDs scores across different gastric cancer datasets, with GSE183136 exhibiting the highest expression level. Subsequently, we conducted correlation analysis between COMMDs scores and available clinical features, revealing a significant elevation of COMMDs scores in tumor tissues compared to non-tumor tissues in the TCGA cohort **(Figure 2B)**. The assessment of current COMMDs scores across different datasets indicated associations with various tumor stages, tumor locations, and treatment responses, including gender and receipt of chemotherapy **(Figure 2C)**.

### Pathway Enrichment Analysis of COMMDs Score

**Figure 3:**
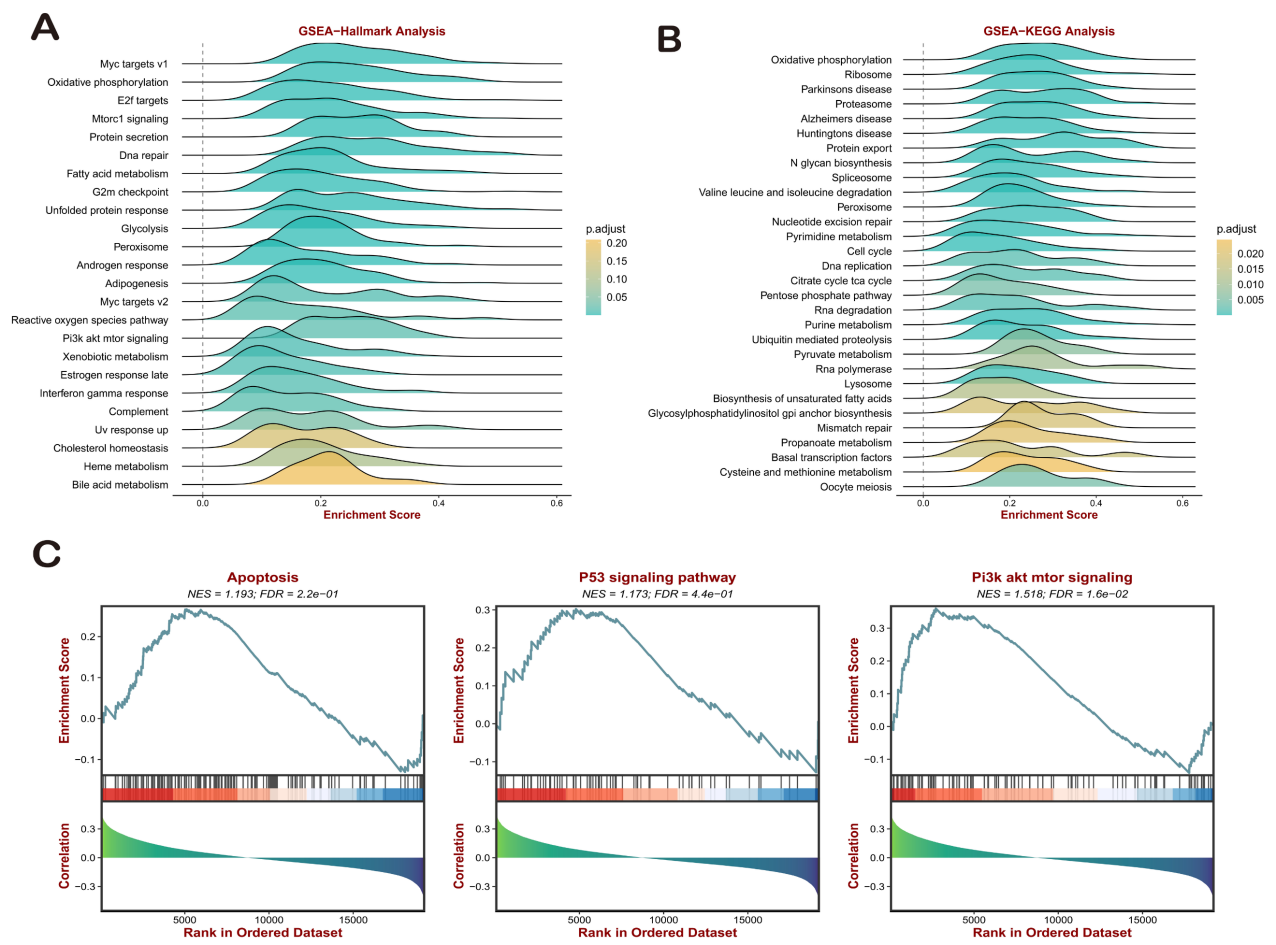
Enrichment Analysis of COMMD Scores. (A) GSEA-HALLMRAKs enrichment analysis of COMMD scores. (B) KEGG enrichment analysis of COMMD scores. (C) Significant GSEA analysis of COMMD scores.

To assess the potential application prospects of COMMDs scores in gastric cancer, we employed GO, KEGG, and GSEA methods to compare various pathways, including hallmarks (**Figure 3A-C, Supplementary Figure 2**). In light of the enrichment in tumor-related pathways, GSEA-hallmark analysis elucidated the positive correlation between COMMDs scores and several classical signaling pathways, including Myc, E2f, Mtorc1, and PI3K-AKT signals. Additionally, a close association with biological activities such as G2M checkpoint, Interferon gamma response, and Complement was observed **(Figures 3A)**. The KEGG results, as shown in **(Figure 3B)**, indicated a close correlation between COMMDs scores and pathways such as Proteasome, Protein export, RNA degradation, Protein processing in endoplasmic reticulum, DNA replication, Mismatch repair, Nucleotide excision repair, and Spliceosome. In order to better understand the potential mechanisms of influence of the COMMDs family in gastric cancer, we have individually demonstrated the currently weighty pathways, which include apoptosis, the P53 pathway, and the PI3K-AKT pathway **(Figure 3C)**.

### Insights into the Immune Orientation of COMMDs Scores in Gastric Cancer Treatment

Further analysis was conducted to determine the correlation between COMMDs scores and the tumor microenvironment (TME). The heatmap clearly suggests varying degrees of correlation between COMMDs scores and immune characteristics across different datasets. This correlation extends to immune cells and immune checkpoint molecules, specifically demonstrating significant correlations with CD8 T cells, Macrophages, Smooth muscle cells, and DC cells, among others. Conversely, NK T cells, CD4 T cells, and Endothelial Cells exhibited insignificant correlations. Analysis of different immune factors also displayed significant heterogeneity, with B2M, CXCL8, CXCL9, CXCL10, among others, showing notable correlations with COMMDs scores **(Supplementary Figure 1 A, B)**.

**Figure 4:**
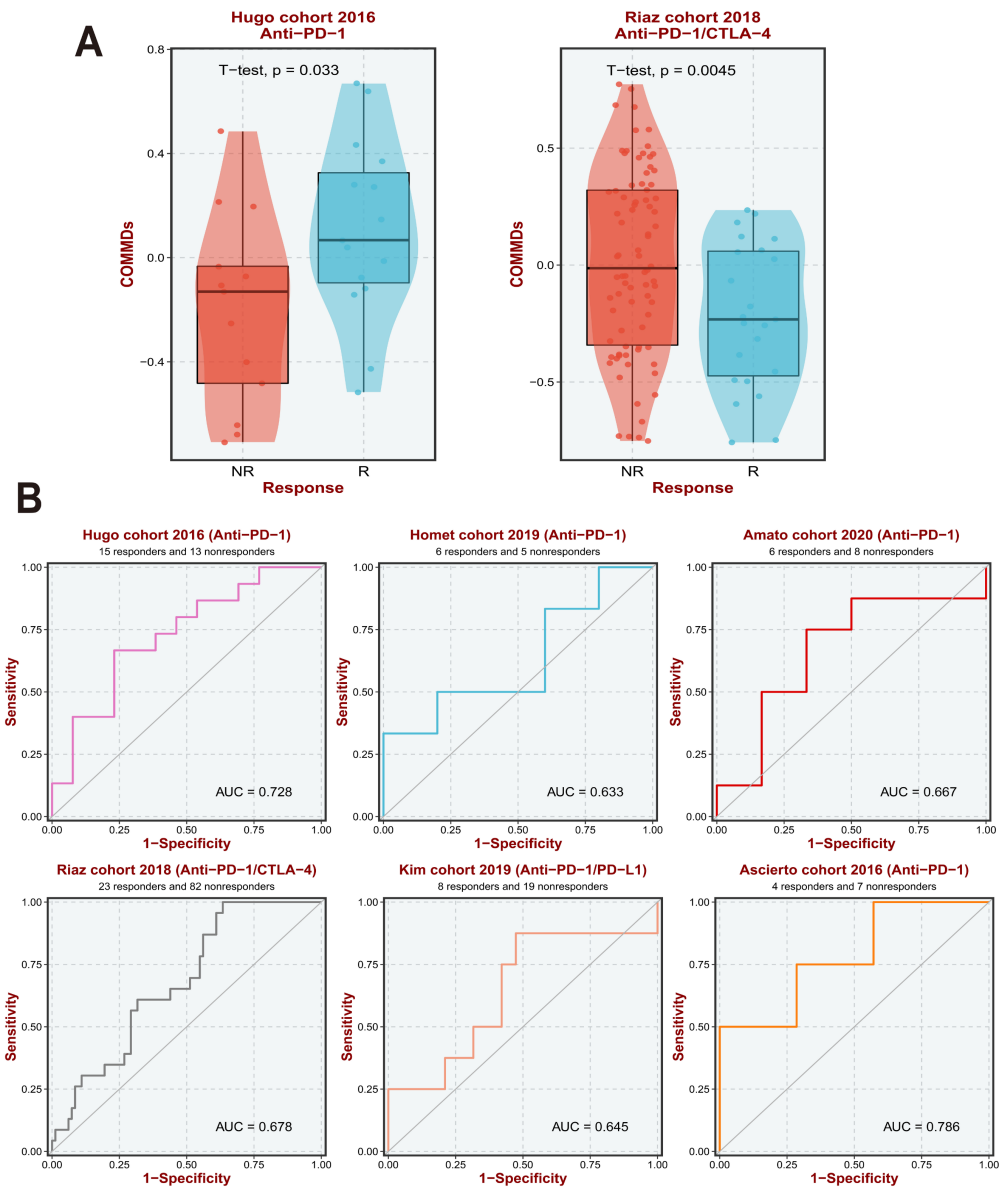
COMMDs Scores are able to predict the effect of immunotherapy. (A) COMMDs Scores can predict the outcome of different immunotherapy treatments. (B) Diagnostic value of COMMDs Scores in different immunotherapy cohorts.

Our research prompted further exploration of the relationship between existing immune therapy datasets and current COMMDs scores. Our findings suggest that a high COMMDs score corresponds to a relatively clear lack of response to immune therapy, with the current score demonstrating good sensitivity across different immune therapy cohorts **(Figure 4A, B)**.

### Single Nucleotide Variations of COMMD Family Genes and their Prognostic Value

**Figure 5:**
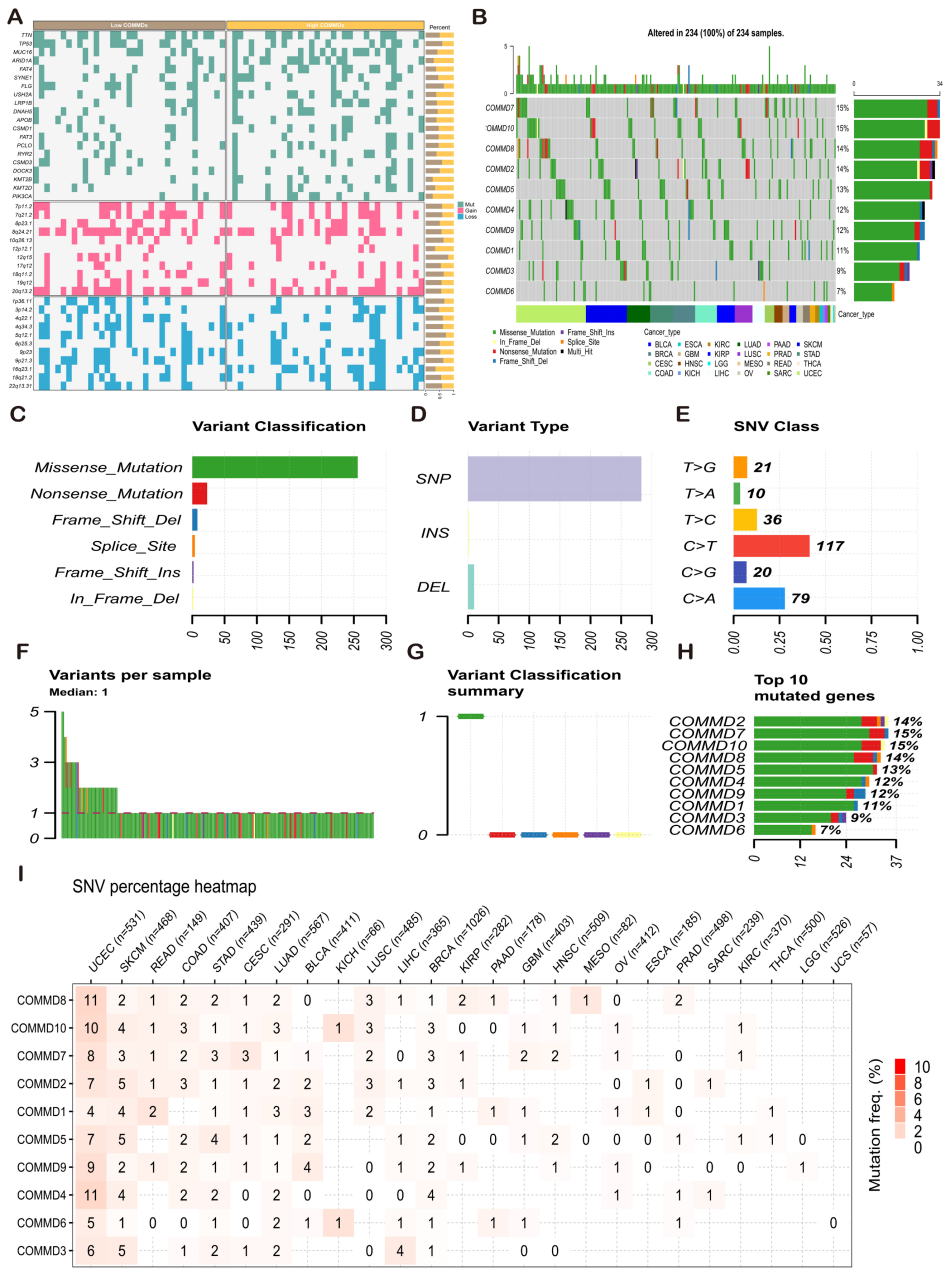
Genetic Mutation Information of COMMD Family Genes. (A) Mutation landscape in populations with high and low COMMD scores. (B) Tumor maps displaying the distribution of FOXO family mutations in pan-cancer tissues. (C-H) Summary of SNVs in the pan-cancer FOXO family. (I) Distribution of SNVs of COMMD family genes in each cancer type.

Mutation and copy number variation data from the TCGA database were processed using MafTools and GISTIC2.0. The statistical differences in mutation and copy number variation ratios between high and low COMMDs score groups are illustrated in **(Figure 5A)**, indicating distinct chromosomal alterations corresponding to different COMMDs score populations. Further exploration of the single nucleotide variation (SNV) landscape of COMMD family genes is presented in **(Figure 5B-I)**. Both COMMD7 and COMMD10 exhibited high-frequency SNV mutations in pan-cancer, predominantly of Missense Mutation type, followed by Nonsense Mutation. In gastric cancer, the most significant mutation information was associated with COMMD5.

### Machine Learning for Identifying the Optimal Model for COMMDs Family Genes

**Figure 6:**
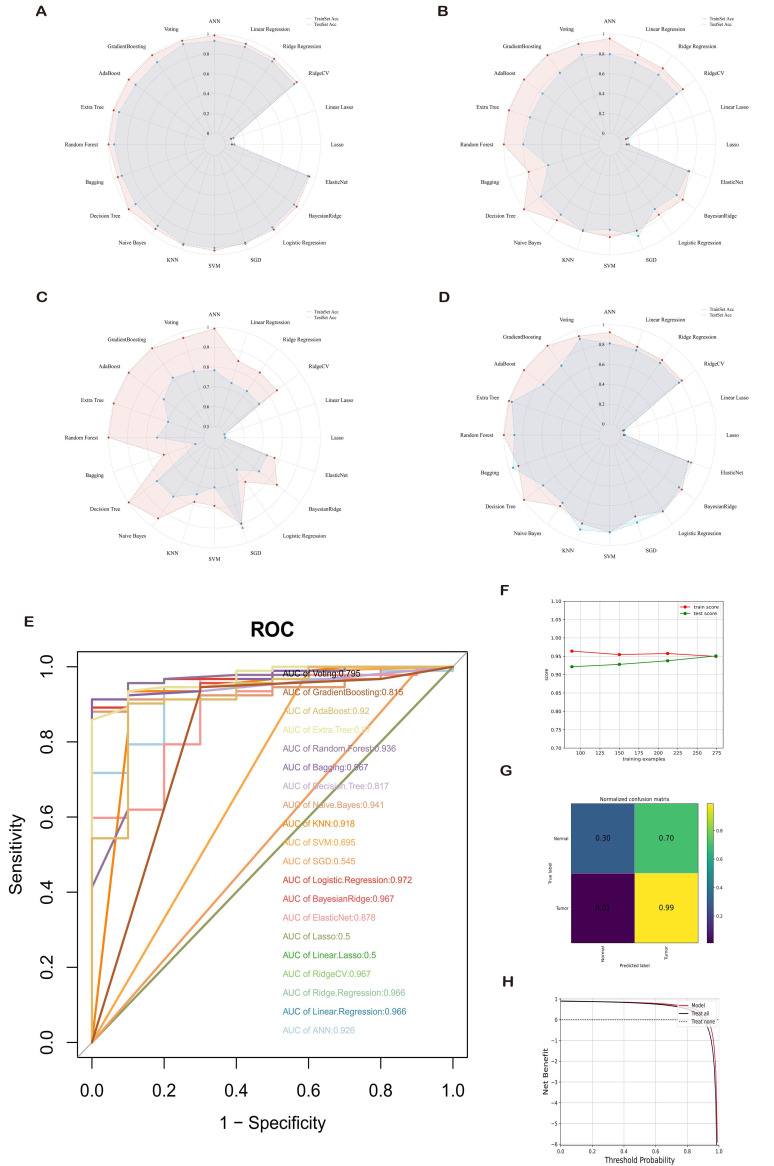
Comprehensive Evaluation of 20 Mainstream Machine Learning Models. (A-D) Radar charts demonstrating accuracy, recall, and F1 measurement on both training and test sets. (E) Receiver Operating Characteristic (ROC) curves comparing the area under the curve (AUC) values for each machine learning model. Generally, an AUC value exceeding 0.7 is considered good predictive performance. (F-H) Performance evaluation of the optimal diagnostic model.

Comparisons were made across various aspects, including accuracy, precision, recall, and F1 measurement, to determine the optimal machine learning model. Overall, when using ROC-AUC values as a criterion, Logistic Regression (0.972), ExtraTree (0.97), Bayesian Ridge (0.967), etc., emerged as top-performing algorithms **(Figure 6A-E)**. Further evaluation of the DCA curve and learning curve for Logistic Regression is presented in **(Figure 6F-H)**, demonstrating stable and excellent learning performance in the training set, with differences between training and test scores being less than 10%. Consequently, the model is considered to possess high generalization capability.

### COMMD10 Promotes Gastric Cancer Progression Through the Mtor-Pi3k-Akt Pathway

**Figure 7:**
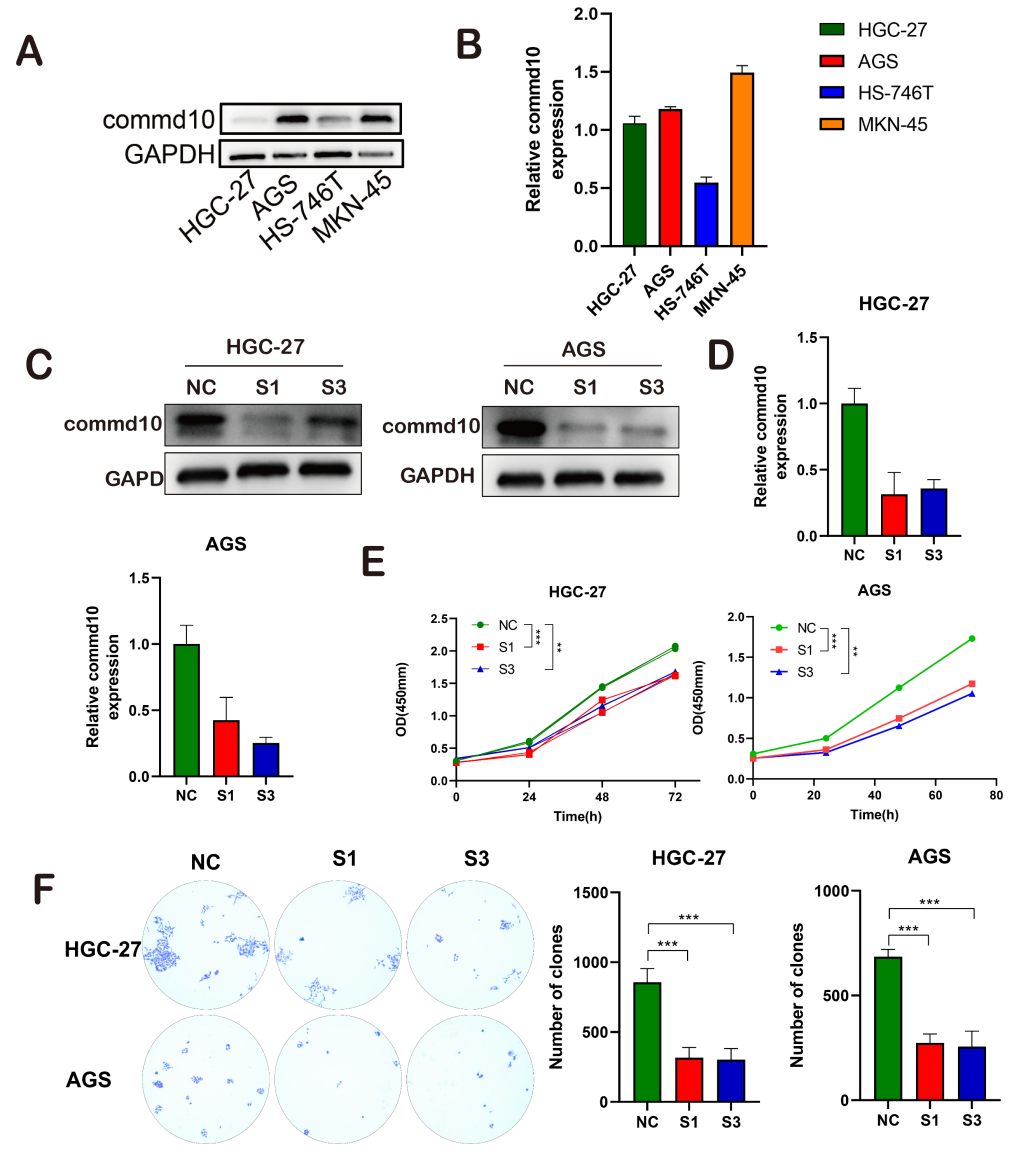
Impact of COMMD10 Gene Interference on Proliferation, Invasion, and Migration of Gastric Cancer Cells. (A-C) Successful downregulation of COMMD10 expression. (E) CCK-8 assay assessing the proliferation capacity of gastric cancer cells. (F) Colony formation assay comparing the impact of COMMD10 on cell proliferation.

**Figure 8:**
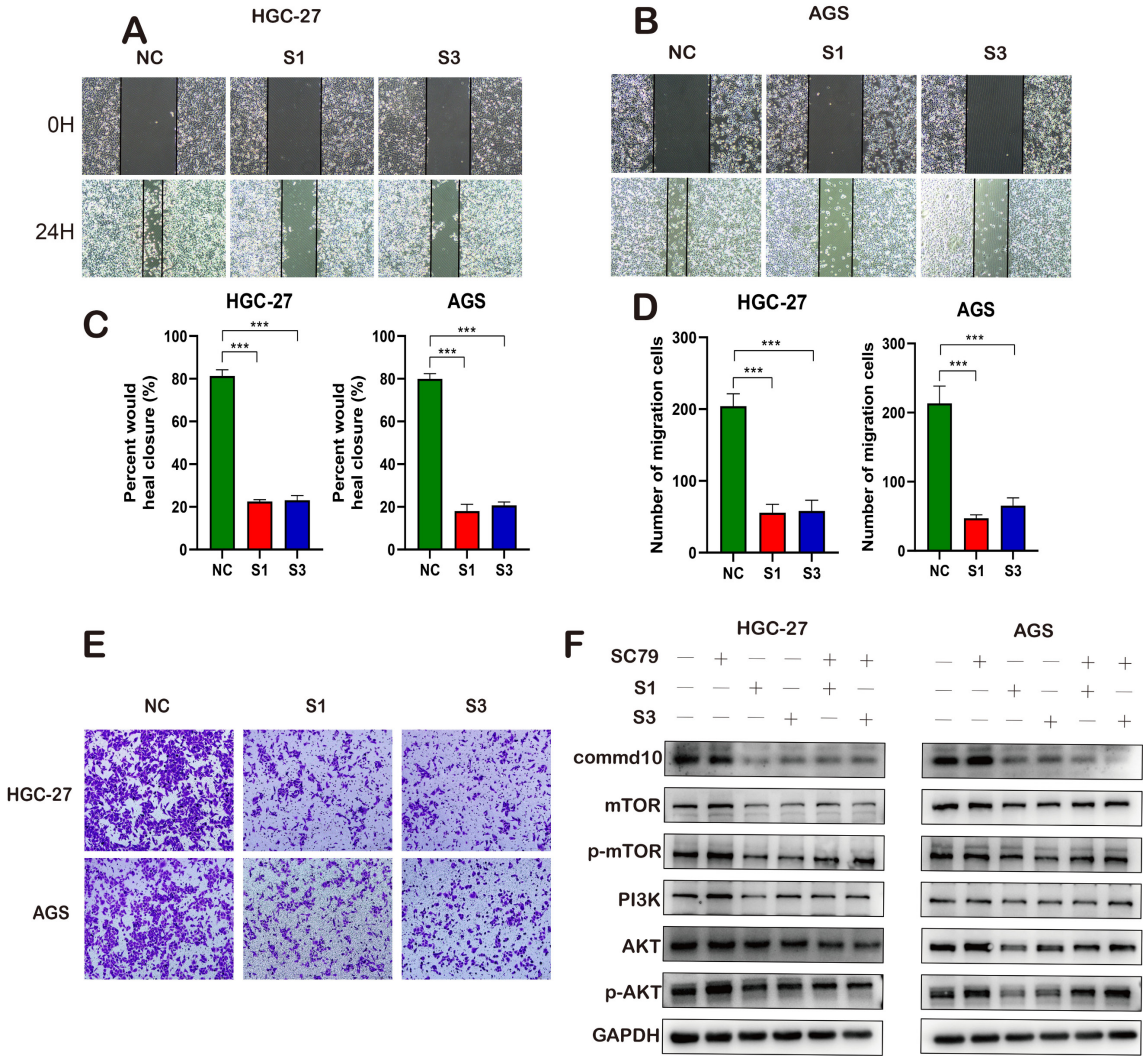
Functional Effects of COMMD10 on Gastric Cancer Cells and its Mechanisms. (A-B) Cell scratch experiment detecting cell migration ability. (C, D) Tranwell assay assessing cell migration and invasion capabilities. (I) Western blotting detecting the impact of interfering with COMMD10 gene expression on the PI3K pathway.

Given the indication of poor prognosis in gastric cancer patients with high COMMD10 expression, we conducted further molecular biology experiments to validate the potential mechanism of COMMD10. As illustrated in **(Figure 7A, B, C, D)**, first we used four cell lines to verify the expression of COMMD10 gene in gastric cancer cell lines, which was consistent with our results in https://depmap.org, so we randomly selected HGC-27 and AGS cell lines for subsequent experiments, we designed two siRNA fragments for expression knockdown in HGC-27 and AGS cells, resulting in varying degrees of changes at both the RNA and protein levels for COMMD10. Subsequent CCK8 and colony formation assays confirmed a significant decrease in the vitality of gastric cancer cells after silencing the target gene **(Figure 7A-D)**. Scratch and migration assays further corroborated the initial findings** (Figure 8A-E)**. To delve into the potential mechanism of COMMD10, we performed WB assays, revealing that downregulating the target gene COMMD10 led to the inhibition of the Mtor-Pi3k-Akt pathway. The expression levels of P-Mtor and P-Akt also decreased, and upon the addition of the AKT pathway activator SC79, the expression levels of P-Mtor and P-Akt were upregulated. This suggests that SC79 can attenuate the inhibitory effect of COMMD10 knockdown on the phosphorylation expression of key proteins in the AKT pathway of gastric cancer cells **(Figure 8F)**.

## Discussion

As of today, it can be affirmed that the formation and progression of tumors involve a complex process with multiple steps and stages. In detail, alterations such as mutations, deletions, overexpression, and epigenetic regulation of key genes occur in various pathological stages of tumors, including decreased apoptosis, unlimited proliferation, interactions with surrounding stromal cells, and eventual distant metastasis 29-31. To precisely prevent and treat cancer, comprehensively understanding the mechanisms of tumor-specific relevant genes or different metabolic characteristics of tumors is a necessary measure to unveil the mystery of cancer 31, 32. Whether taking an unconventional path or returning to basics, based on the physiological knowledge that the human body's copper levels are in dynamic equilibrium, disruptions in both hereditary and acquired copper homeostasis can lead to or exacerbate certain diseases. Researchers have begun exploring the possibility of targeting copper metabolism. Copper, as an essential trace element in the human body, not only plays a crucial role as a co-factor in redox reactions due to its two ionic states but is also an important component of many metabolic key enzymes, including Cu/Zn superoxide dismutase 1 (SOD1), cytochrome c oxidase (CCO), and others 33. Recent discoveries, such as copper death (cuprotosis), further emphasize the body's need for copper homeostasis mechanisms 34, 35.

The COMMD family plays a crucial role in regulating local and systemic metabolism of cells and organisms in copper control, especially in the field of cancer research. COMMD1, as the star molecule of the COMMDs family, has been confirmed to be involved in regulating the invasion, metastasis, and chemo-resistance of malignant tumors such as neuroblastoma, head and neck squamous cell carcinoma, lung cancer, colorectal cancer, glioblastoma, and melanoma cells 36, 37. Genes like COMMD5, COMMD7, and COMMD9 have also been confirmed to promote the dephosphorylation of ErbB2/HER2 or downstream molecules, such as PIAS or CXCL1, to participate in the regulation of non-small cell lung cancer, hepatocellular carcinoma, renal cell carcinoma, and pancreatic ductal adenocarcinoma 37. However, there is scarce research on the COMMDs family genes in gastric cancer, and our study fills in this gap, providing further clarification of the potential anti-gastric cancer mechanisms of this gene family. Based on existing transcriptomic data resources, we examined the expression and prognostic value of the current ten COMMDs genes in gastric cancer. In comparison to COMMD1/6, the other COMMDs genes exhibited a significant trend of high expression in gastric cancer. COMMD1 captured our attention, as mentioned earlier, it has been confirmed to play a regulatory role in various cancers, indicating its intrinsic regulatory value. The current RNA-level differences may not entirely hinder its prospective application in gastric cancer, and future in-depth research on its protein level or exploration in areas such as transcriptional regulation or protein modification may uncover different aspects. This is based on our in-depth exploration of prognostic value because among many COMMDs family genes, COMMD1/10 showed significant prognostic value. Future in-depth studies on these two genes may inspire targeted therapies for gastric cancer.

COMMD10, in contrast to COMMD1, has been extensively studied in the context of liver cancer. A prior study by Yang *et al.* identified COMMD10 as a gene associated with the radiosensitivity of liver cancer within the COMMD gene family. Subsequent research demonstrated that COMMD10 regulates radiation resistance in liver cancer cells by reducing intracellular copper levels and inducing ferroptosis 38. Moreover, COMMD10 exhibits molecular biology mechanisms in colorectal cancer similar to COMMD1, blocking the activation of the NF-κB pathway by reducing p65 nuclear translocation, thereby inhibiting the invasion and metastasis of colorectal cancer 39, 40. Beyond these studies, COMMD10 has been shown to inhibit both typical and atypical inflammasome activities of Ly6Ch monocytes in a mouse model of lipopolysaccharide-induced inflammation, alleviating the damage caused by inflammatory storms 41, 42. Our analysis of COMMDs family genes suggested that COMMD10 might be a crucial molecule influencing the malignant progression of gastric cancer. Subsequently, our intervention in the gene expression levels of gastric cancer cells successfully confirmed our initial conclusion. Moreover, similar to the earlier enrichment analysis suggestions, the COMMDs score showed a significant correlation with the activation of the PI3K pathway, a relationship confirmed by our current western blot experiments. Considering our study involves tumor immunity and previous research on the impact of the PI3K pathway on immunotherapy, we contemplate the future potential application of COMMD10 in immunotherapy, which requires further investigation 43. In comparison to Wang *et al.*'s previous study, our focus is more on the COMMDs family genes as a whole. We then explored the translational medical value of the developed COMMDs score at different levels 44. Similar to each COMMDs family gene, the COMMDs score showed a positive correlation with the malignancy of gastric cancer, with higher COMMDs scores in tumor tissues than in non-tumor tissues. Additionally, the COMMDs score was related to clinical characteristics such as tumor staging, tumor location, gender differences, and chemotherapy treatment. However, a limitation of our study is that the conclusions are drawn from different gastric cancer transcriptomic expression profiles, and future large-sample studies may provide further validation. Moreover, we expanded the application scope of the COMMDs score, and different enrichment analysis methods suggested a common index: the COMMDs score might influence the direction of immunotherapy, including the involvement of Myc, E2f, Mtorc1, and PI3K-AKT signals in the transformation of the tumor immune microenvironment. Given that immunotherapy seems to be a significant measure for gastric cancer, we assessed the relationship between the COMMDs score and immune cells or immune factors, indicating that individuals with high COMMDs scores may exhibit unresponsiveness to immunotherapy. We tested this effect in different immunotherapy cohorts, and the results suggested good efficacy of the current COMMDs score 45. The preliminary efficacy of the current COMMDs score prompted us to broaden its translational medical value through machine learning algorithms. As mentioned earlier, the COMMDs score may become a biomarker for diagnosing gastric cancer patients or evaluating the macroscopic status of the tumor microenvironment (TME). It may also serve as a potential prognostic marker for predicting various treatments, especially immunotherapy efficacy.

In conclusion, building on the wide-ranging biological roles of the COMMD protein family established in previous research, our study, employing various methods, has demonstrated that the COMMDs family is not only involved in the proliferation and invasion of gastric cancer but also acts as a therapeutic target and a biomarker for evaluating the prognosis and staging of tumors. Further research into the functions of COMMD proteins in tumors contributes to the clinical diagnosis and treatment of cancer.

## Supplementary Material

Supplementary figures.

## Figures and Tables

**Table 1:** Role of the COMMDs family in tumor proliferation, migration, invasion, and immunotherapy

Commds	Cancer type	Role	Regulated target	Refs.
Commd1	Melanoma	Tumor suppressor	HIF-1α/β	46
	Head and neck squamous cell carcinoma	Tumor suppressor	NF‑κB/ TNFR/IL-1R	13
	Non-small cell lung cancer	Tumor suppressor	NF‑κB/ TNFR/IL-1R	13
Commd2	Bladder cancer	Oncogene	PI3K/AKT/ mTOR	47
	Hepatocellular carcinoma	Oncogene	PD-1/PDL/CTLA-4	48
Commd3	Hepatocellular carcinoma	Oncogene	HIF1α/VEGF/NF-κB	15
	Breast cancer	Tumor suppressor	ATP1B1	16
Commd4	Glioma	Oncogene	MC	49
	Non-small cell lung cancer	Oncogene	H2B/PPI	8
Commd5	Renal cell carcinoma	Tumor suppressor	HCaRG/ErbB	50
Commd6	Unverified	Unverified	Unverified	*
Commd7	Hepatocellular carcinoma	Oncogene	NF-κB/ Nanog^+^HCSCs	14
	Pancreatic Ductal Adenocarcinoma	Oncogene	MEK1/2-ERK1/2	51
Commd8	Non‐small cell lung cancer	Oncogene	SP1	17
	Hepatocellular carcinoma	Oncogene	MNX1-AS1	19
Commd9	Non-small cell lung cancer	Oncogene	TFDP1/E2F1	21
Commd10	Hepatocellular Carcinoma	Tumor suppressor	NF-κB/ HIF1α/CP	22, 52
	Colorectal cancer	Tumor suppressor	NF-κB/ FMNL2	23
	Gastric cancer	Oncogene	M6A/STAD	53
