# Supplementary material for: Decoding Gastric Cancer: Machine Learning Insights Into the Significance of COMMDs Family in Immunotherapy and Diagnosis
Source: J Cancer. 2024 May 11;15(11):3580–95. doi: 10.7150/jca.94360 (PMC11134438; doi:10.7150/jca.94360)
Supplement: Supplementary file 1 — Supplementary figures. [file jcav15p3580s1.pdf]

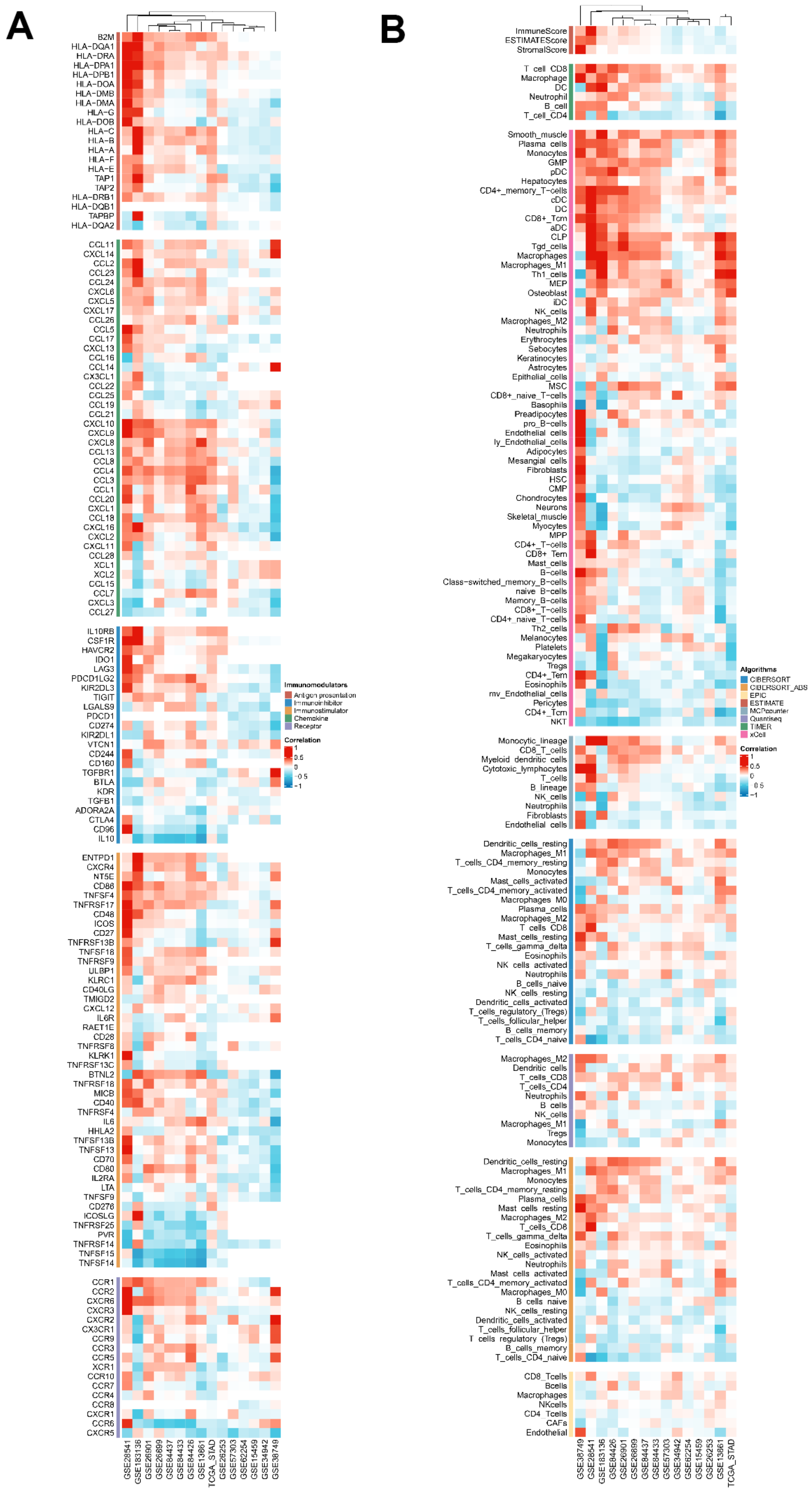


**Supplementary Figure 1:** Correlation Analysis of COMMD Scores with Tumor Immune-Related Features. (A) Heatmap illustrating scores of different immune cells in the tumor microenvironment calculated by various algorithms. (B) Heatmap depicting the correlation between COMMD scores and immune factors.

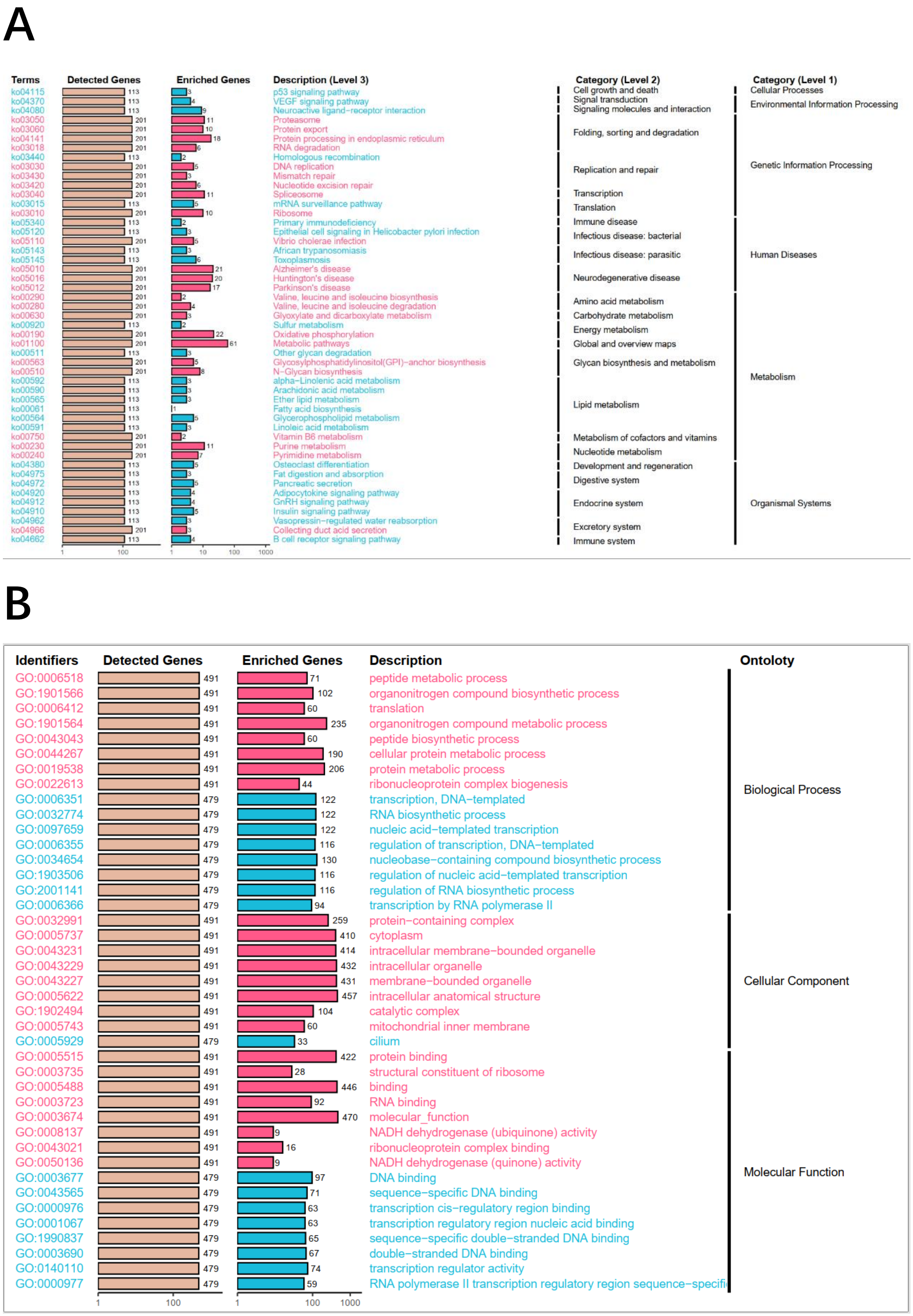


**Supplementary Figure 2:** Enrichment Analysis of COMMD Scores. (A) KEGG enrichment analysis of COMMD scores. (B) GO enrichment analysis of COMMD scores.
